# Metformin Counteracts HCC Progression and Metastasis Enhancing KLF6/p21 Expression and Downregulating the IGF Axis

**DOI:** 10.1155/2019/7570146

**Published:** 2019-01-10

**Authors:** Fernanda Vacante, Pamela Senesi, Anna Montesano, Stefano Paini, Livio Luzi, Ileana Terruzzi

**Affiliations:** ^1^Metabolism Research Center, IRCCS Policlinico San Donato, San Donato Milanese, Milan, Italy; ^2^Department of Biomedical Sciences for Health, Università degli Studi di Milano, Milan, Italy

## Abstract

**Background and Aims:**

Hepatocellular carcinoma (HCC) is the common tumor of the liver. Unfortunately, most HCC seem to be resistant to conventional chemotherapy and radiotherapy. The poor efficacy of antitumor agents is also due, at least in part, to the inefficient drug delivery and metabolism exerted by the steatotic/cirrhotic liver that hosts the tumor. Thus, novel approaches in chemotherapy may be needed to improve the survival rate in patients with HCC. Metformin (METF) has been found to lower HCC risk; however, the mechanisms by which METF performs its anticancer activity are not completely elucidated. Previous studies have showed METF action on growth inhibition in the liver in a dose/time-dependent manner and its antitumor role by targeting multiple pathways. We investigated molecular effects of METF in an *in vitro* human hepatoma model (HepG2), studying cell cycle regulators, tumorigenesis markers, and insulin-like growth factor (IGF) axis regulation.

**Materials and Methods:**

HepG2 cells were treated with METF (400 *μ*M) for 24, 48, and 72 hours. METF action on cell cycle progression and cellular pathways involved in metabolism regulation was evaluated by gene expression analysis, immunofluorescence, and Western blot assay.

**Results:**

By assessing HepG2 cell viability, METF significantly decreased growth cell capacity raising KLF6/p21 protein content. Moreover, METF ameliorated the cancer microenvironment reducing cellular lipid drop accumulation and promoting AMPK activity. The overexpression of IGF-II molecule and the IGF-I receptor that plays a main role in HCC progression was counteracted by METF. Furthermore, the protein content of HCC principal tumor markers, CK19 and OPN, linked to the metastasis process was significantly reduced by METF stimulus.

**Conclusion:**

Our data show that METF could suppress HepG2 proliferation, through induction of cell cycle arrest at the G0/G1 phase. In addition, METF effect on the cancer microenvironment and on the IGF axis leads to the development of new METF therapeutic use in HCC treatment.

## 1. Introduction

Hepatocellular carcinoma (HCC) is a significant health issue, particularly in developing countries, where it is inevitably fatal: it represents the third most frequent cause of cancer-related death, with an annual incidence of 700,000 new cases [1]. Half of HCC cases occur in China, where B and C hepatitis are the major risk factors for HCC [1]. The other 30–40% of cases, which occurs in Western countries, are probably attributable to nonalcoholic fatty liver disease (NAFLD) or metabolic syndrome [2]. Indeed, epidemiologic studies have shown that diabetes mellitus (DM), with insulin resistance, is an independent risk factor for HCC and it is positively associated with increased risk of liver cancer [2].

Though, in recent years, the diagnosis of HCC is largely improved as well as the management of the early stage, the prognosis of liver cancer remains extremely poor [1, 3]. In most cases, chemotherapy and radiotherapy have palliative effects. Reducing the risks or delaying the onset of HCC represent promising chemopreventive strategies.

HCC metastases and invasiveness are significant causes of cancer-related morbidity and mortality [4], and metastasis incidence is expected to decrease with the improvements of systemic therapies. Recent works have showed the importance of the microenvironment for tumor initiation and development, including metabolic alterations, growth, and metastasis [5]. However, the mechanisms of metastatic cascade remain poorly understood [6]. Cytokeratin-19 (CK19) expression was correlated with metastasis, early tumor reappearance after resection, and radiofrequency ablation. Indeed, the CK19 knockdown cells significantly reduced HCC invasive ability instead; human CK19-positive tumor cells showed increased invasiveness [7].

Recent works have showed the importance of the microenvironment for tumor initiation and growth and for the development of metabolic alterations and metastasis [6, 8]. Chronic inflammation is intimately associated with the pathogenesis of HCC and metastatic formation [9].

Osteopontin (OPN), secretary phosphorylated glycoprotein, plays a key role in promoting the metastatic process in HCC [10]. In fact, OPN, involved in various pathological conditions including inflammation and angiogenesis, is secreted by malignant cells in advance metastatic cancer and induces epithelial-mesenchymal transition with upregulation of the mesenchymal markers and E-cadherin downregulation [11].

Moreover, annexin A5, a calcium- and phospholipid-binding protein, is generally used to detect apoptosis phenomena. Nonetheless, there is recent evidence that annexin A5 is related to the efficacy of cancer therapies. In particular, Jeong et al. have demonstrated that annexin A5 plays an important role as a mediator of cisplatin antitumorigenesis action [12]. Recently, Li et al. have demonstrated that annexin A5 overexpression in the uterine cervical carcinoma might negatively regulate cell proliferation [13]. Thus, these studies suggest how induction of annexin A5 could be a promising target in cancer therapy.

IGF axis components, as the insulin-like growth factors I and II (IGF-I and IGF-II) and their receptors, are implicated in tumor formation, growth, and metastasis *in vivo*. In particular, a crucial role has been attributed to the IGF-I receptor, which mediates mitogenic signals, and it is necessary for the malignant transformation of certain types of cells [14].

Metformin (METF) is the most commonly used drug in the treatment of type 2 diabetes, introduced into clinical practice in the 1950s [15]. It reduces the risk of hyperglycemia predominantly by decreasing hepatic glucose production via inhibition of gluconeogenesis, enhancing glucose uptake, and utilization by reversal of insulin resistance in peripheral tissues [15]. The glucose-lowering action of METF is reliant on liver kinase B1- (LKB1-) dependent activation of adenosine monophosphate-activated protein kinase (AMPK), a conserved cellular energy sensor that is activated in response to an increased AMP/ATP ratio caused by various cellular stresses [16]. Moreover, it has been shown that METF-mediated activation of AMPK increases fatty acid oxidation in the liver [15, 17], decreasing lipogenesis [18] through the inhibition of specific enzymes, transcription factors, and malonyl CoA's activation [19, 20].

Interestingly, several works suggest that the central therapeutic properties of METF are regulated independently by the AMPK pathway [20, 21].

A surprising finding in recent articles is that METF might be useful in the prevention and treatment of several common cancers [22, 23]: it acts through both insulin-dependent [24] and insulin-independent mechanisms [25]. Epidemiological studies have shown that METF, used among diabetic patients, resulted in a 50% risk reduction in HCC incidence [22, 26]. On the other hand, insulin and insulin secretagogues have been associated with a 62% increase and a 161% increase of incidence and cancer-related mortality [27].

However, the mechanisms by which metformin performs its anticancer activity are not completely explained.

Recent *in vitro* and *in vivo* studies have shown that in the liver, METF is able to inhibit selectively the growth of cancer cells [28], without action of normal hepatocytes, in a dose- and time-dependent manner [29]. The METF-activated AMPK could contribute to inhibitory effects of METF in HCC cells [30, 31], even if several authors propose an AMPK-independent drug effect [20, 32].

Surely, METF acts on the main regulators of the cell cycle, as cyclin, cyclin-dependent kinases (CDKs), and CDK inhibitors (CDKIs), by blocking the cells in the G0/G1 phases [30, 32]. p21^CIP1^ and p27^KIP1^ can prevent inappropriate cyclin/CDK activity in the G1 phase [33]. Moreover, p53, a tumor suppressor and an upstream regulator of p21^CIP1^, can indirectly affect the cell cycle [33]. These mechanisms, associated with the control of restriction point, are usually impaired in cancer cells. Hence, the repair of uncontrolled cell cycle progression might be an effective strategy for the treatment of HCC.

Many *in vitro* and *in vivo* studies have already shown that METF could exert its antitumor effect by targeting multiple pathways such as cell cycle/apoptosis, AMPK/mTOR, anti-inflammatory pathway, insulin/IGF-IR, and angiogenesis.

However, because the dosage of METF used in these studies (1–20 mM) was much higher than the dose used in the treatment of diabetic patients, the aim of this study was to describe the *in vitro* effects of human therapeutic concentration of METF (400 *μ*M) in liver cancer cells. In particular, we have investigated the action of the drug on the major proteins regulating cell cycle, on tumorigenesis marker synthesis, and on IGF/insulin axis regulation.

## 2. Materials and Methods

### 2.1. Chemicals and Reagents

Metformin (1,1-dimethylbiguanide) was purchased from Sigma (Sigma Chemical Co., Saint Louis, MO, USA). All primary antibodies: calnexin (H-70), GAPDH (FL-335), annexin A5 (R-20), AMPK*α*1/2 (H-300), cytokeratin 19 (N-13), IGF-IR*β* (C-20), KLF6 (R-173), OPN (K-20), PGC-1*α* (H-300), p53 (FL-393), p21 (C-19), Rb (C-15), pRb (Ser249/Thr252), peroxidase-conjugated econdary antibodies for Western blot analysis, and Rhodamine/FITC-conjugated antibodies for immunofluorescence analysis were obtained from Santa Cruz Biotechnology (Santa Cruz, CA, USA).

Primary antibody p-AMPK*α* (Thr 172) was purchased from Cell Signaling Technology (Danvers, MA, USA).

### 2.2. Cell Lines and Culture Conditions

**Figure 1:**
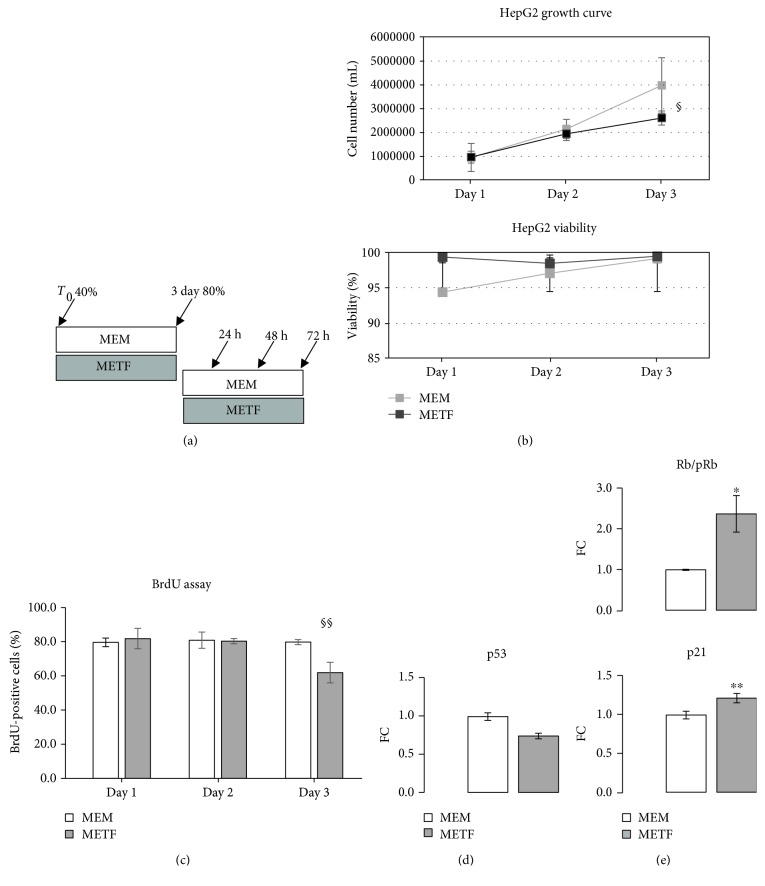
Metformin action on the proliferative phase of HepG2 cells: (a) experimental scheme of HepG2 treatments; (b) growth curve and viability determination: treatment with 400 *μ*M METF significantly decreased the proliferative capacity of HepG2 without inducing cell death; (c) BrdU incorporation assay: METF did not suppress HepG2 cell proliferation at 24 and 48 h while at 72 h of treatment, METF significantly induced a proliferation decrease. Representative images related to BrdU assay were added as supplementary data (available here); (d) to confirm the results obtained by the growth curve, we analyzed p53 protein content: the protein content of p53 at day 3 of the growth curve decreased with respect to that of control in the presence of 400 *μ*M METF; (e) METF significantly risen Rb activation. Conversely, the p21 protein level was increased in response to 72 h METF treatment. Data are expressed as fold change (FC) mean ± SD. Representative Western blots were added as supplementary data. Significance: *t*-test: ^∗^ *p* ≤ 0.05 vs MEM, ^∗∗^ *p* ≤ 0.01 vs MEM. For growth curve, viability, and BrdU assay, ANOVA test followed by Sidak's multiple comparison test was used. ANOVA test: §*p* ≤ 0.05 and §§*p* ≤ 0.01.

Human hepatocellular carcinoma cell line HepG2 was obtained from the European collection of cell cultures (ECACC) and maintained in MEM containing 10% fetal bovine serum (FBS), 1% penicillin streptomycin, 1% glutamine, and 1% of nonessential amino acids. The cells were incubated in a humidified atmosphere of 5% CO_2_ at 37°C and passaged by trypsinization when they reached 80% confluence. The culture medium was changed every day, following literature indications. For experiments, HepG2 cells were treated with METF 400 *μ*M as indicated in the legend of Figure 1.

### 2.3. Growth Curve and Cell Viability Test

HepG2 cells (2 × 10^5^) were plated on 60 mm × 15 mm culture dishes at 40% confluence and grown in MEM. The cells were treated or not with METF 400 *μ*M (Figure 1(a)). At 24, 48, and 72 h after treatment, cells were trypsinized, stained with trypan blue, and counted using hemocytometer. The average values for each single day were used to plot a growth curve. Cell viability was calculated by dividing the nonstained viable cell count by the total cell count. In addition, morphological changes were examined daily by phase contrast microscopy.

#### 2.3.1. 5-Bromo-2′-deoxyuridine (BrdU) Assay

HepG2 cells (1.5 × 10^5^) were seeded on 60 mm × 15 mm culture dishes and grown in MEM. The cells were treated or not with METF 400 *μ*M for 24, 48, and 72 h. Briefly, BrdU solution was added to each well and cells were incubated at 37°C for 3 h, in a cell culture incubator protected from light. Cell medium was removed and cells were fixed with 4% paraformaldehyde for 15 min. Cells were permeabilized with 0.3% Triton X-100 in PBS for 10 min and blocked with 5% bovine serum albumin in PBS with 0.2% Triton X-100 solution for 1 h. Cellular DNA was denatured by DNase RQ1 (Promega, France) in PBS with 0.2% Triton X-100 solution. The incorporated BrdU was stained with anti-BrdU monoclonal antibody (Sigma Chemical Co., Saint Louis, MO, USA). Nuclei were revealed with DAPI staining. Cell images of each treatment were captured with Nikon Eclipse 50I microscopy. Automated quantification signal was performed by using ImageJ program (http://imagej.nih.gov/ij/).

### 2.4. Real-Time PCR (RT-PCR) Analysis

HepG2 (1 × 10^6^) cells were cultured in 100 mm dishes and treated with METF 400 *μ*M for 24 h. Total RNA was extracted from human hepatocellular carcinoma cells HepG2 with RNeasy Plus Mini QIAGEN Kit (Qiagen GmbH, Germany) according to the manufacturer's instructions. The quantity of the RNA in the extraction was determined by measuring the absorbance ratio of A260 and A280 using a spectrophotometer (NanoDrop 8000). The prepared RNA was then reversely transcribed to single-stranded cDNA using RT/PCR kit (GoScript, Promega, France), and it is used as template for analysis of gene expression level.

The sequence of primers to determine the expression of the target gene was listed as follows:

hIGF-II: 5′-CTTCCAGACACCAATGGGAAT-3′, 3′-GTCCCCACAGACGAACTGGA-5′, hIGF-IR: 5′-CTAAACCCGGGGAACTACACAG-3′, 3′-TTCACAGAGGCATACAGCAC-5′, hIGF-IIR: 5′-TACAACTTCCGGTGGTACACCA-3′, 3′-CATGGCATACCAGTTTCCTCCA-5′, hGAPDH: 5′-CGAGATCCCTCCAAAATCAA-3′, 3′-TTCACACCCATGACGAACAT-5′.

The PCRs consisted of 10 minutes at 95°C, followed by 40 cycles of denaturation for 10 seconds at 95°C, annealing, and primer extension for 1 minute at 60°C. All measurements were performed in triplicate. The comparative CT method was used to quantitate the expression of genes using GAPDH as the normalized control. The expression level of the housekeeping genes chosen for normalization in the threshold cycle (Ct) for each experimental condition and then the fold change (ΔΔCt) for each gene from the treatment group compared to the control group was calculated. If the ΔΔCt is greater than 1, then the result may be reported as a fold upregulation. If the ΔΔCt is less than 1, then the result may be reported as a fold downregulation.

### 2.5. Western Blot Analysis

Western blot analysis was performed as described previously [34]. HepG2 cells were grown in 100 mm culture dishes with or without METF. Cell extracts were prepared by lysing the cells in RIPA buffer. 30 *μ*g of protein was separated by SDS-polyacrylamide gel electrophoresis (SDS-PAGE) and electrophoretically transferred to nitrocellulose membranes (Protran®, Whatman® Schleicher & Schuell). The blots were then blocked and incubated with specific primary antibodies, followed by incubation with anti-species-specific secondary antibodies. To confirm equal protein loading per sample, we used anti-calnexin or anti-GAPDH antibody. Finally, detection of specific proteins was performed by enhanced chemiluminescence reagent (Western Lightning ECL Pro, PerkinElmer). Quantitative measurement of immunoreactive band intensities was performed by densitometric analysis using the Scion Image software (Scion Corporation, Frederick, MD, USA). Data were then converted into fold changes (FC) of the control.

### 2.6. Immunofluorescence

HepG2 cells were grown on coverslips with or without 400 *μ*M METF. After 24, 48, and 72 hours of treatment, cells were washed 3 times with PBS, then fixed in prepared 4% paraformaldehyde for 20 minutes, and raised three times in PBS. The cells on the coverslips were washed with PBS and incubated for 30 minutes at room temperature with 1% bovine serum albumin in PBS with 0.2% Triton X-100. Then HepG2 cells were incubated with primary antibodies for 150 minutes. To detect primary antibody, binding site cells were washed three times in PBS and followed by incubation with specific rhodamine/FITC-conjugated antibodies for 90 minutes. Nuclei were revealed with DAPI staining. Coverslips with cells were mounted and observed using Nikon Eclipse 50I microscopy. The images were captured using NIS-Elements D 4.00 software. Data were analyzed using Adobe Photoshop CS4.

### 2.7. Oil Red O Coloration

This technique is based on the staining of intracellular lipids by Oil Red O (Sigma Chemical Co., Saint Louis, MO, USA). HepG2 cells were fixed in prepared 4% paraformaldehyde for 30 minutes at room temperature and washed in distilled water. Then cells were incubated with Oil Red O. solution according to the manufacturer's procedures. The cells on the coverslips were washed in distilled water. Coverslips with cells were mounted and observed by phase contrast microscopy.

Automated quantification signal was performed by using ImageJ program (http://imagej.nih.gov/ij/).

### 2.8. Statistical Analysis

All experiments were performed three times. The data are presented as the mean ± standard deviation, and statistical comparisons were performed with specific statistical packages (Prism v 7.00 GraphPad Software, San Diego, CA, USA). Statistically significant differences were determined using Student's *t*-test or ANOVA test followed by Sidak's multiple comparison test.

Results were considered statistically significant when *p* ≤ 0.05.

## 3. Results

### 3.1. Metformin Treatment Decreases Cell Proliferation and Does Not Induce Cell Death

In order to determine whether 400 *μ*M METF influences human liver cancer cell proliferation, we analyzed the effects of this drug on HepG2, an *in vitro* model of human liver carcinoma.

Cells were cultured in a growth medium with or without METF treatment for three days. As shown in Figure 1(b), 400 *μ*M METF decreased HepG2 cell proliferation and, in this condition, we did not observe cell death: cell viability was not influenced during METF treatment. Using a BrdU incorporation assay, we confirmed that METF did not suppress HepG2 cell proliferation at 24 and 48 h while at 72 h of treatment, METF significantly induced cell cycle arrest (Figure 1(c)).

To corroborate this evidence, by Western blot assay, we evaluated protein content of p53, a key transcriptional factor associated with induction of cell cycle arrest and apoptosis: METF decreased the p53 protein level with respect to control at day 3 of the growth curve (Figure 1(d)).

Based on these data, we hypothesized that 400 *μ*M METF could exert an effect on HepG2 cell growth, leading to a significant decrease in proliferation.

### 3.2. Metformin Treatment Regulates Cell Cycle Proteins

Inappropriate cell cycle progression determines an unlimited cell division in cancer cells. To investigate the molecular mechanisms responsible for the METF-induced cell growth inhibition, Western blot and immunofluorescence assays were used to analyze METF impact on cell cycle-related proteins in HepG2. In proliferating HepG2, the level of phosphorylated Rb was progressively decreased: conversely, the p21 protein level was increased in response to 72 hours of METF treatment (Figure 1(e)).

These *in vitro* data seem to demonstrate that 400 *μ*M METF might affect the expression and the phosphorylation of key proteins of the cell cycle and it leads to an arrest in the G0/G1 phase in hepatocellular cancer cells [30, 32].

**Figure 2 fig2:**
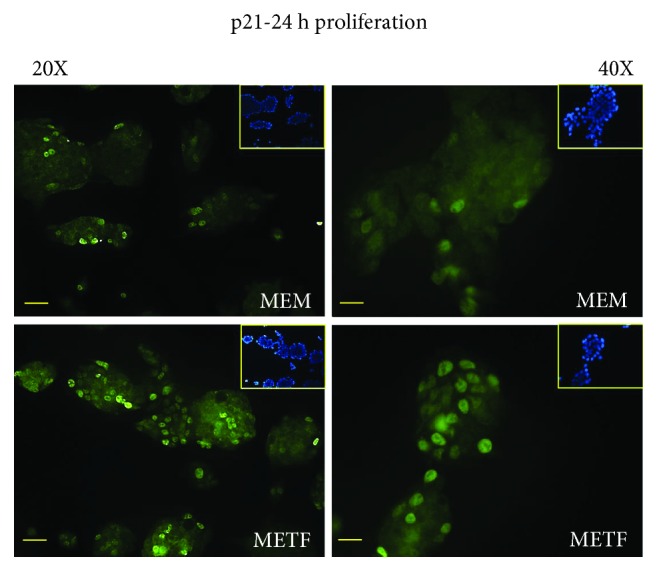
METF action on p21 nuclear translocation: METF stimulated p21 nuclear translocation, confirming that it is able to arrest cell growth by inhibiting cyclin-dependent kinases. These images showed the different localization of the signal in cells treated with METF in respect to MEM control. Scale bars: 200 *μ*m (20X) and 100 *μ*m (40X).

As shown in Figure 2, p21 was mainly localized in HepG2 nuclei after 24 h of treatment. Recent studies show that p21 can act both as a tumor suppressor gene and as an oncogene depending on its cellular localization. When p21 is localized to the nucleus, it arrests cell growth by inhibiting cyclin-dependent kinases and DNA synthesis through interactions with proliferating cell nuclear antigen, PCNA [30].

### 3.3. The Antiproliferative Effect of Metformin Is Not Mediated by p53 Protein

**Figure 3:**
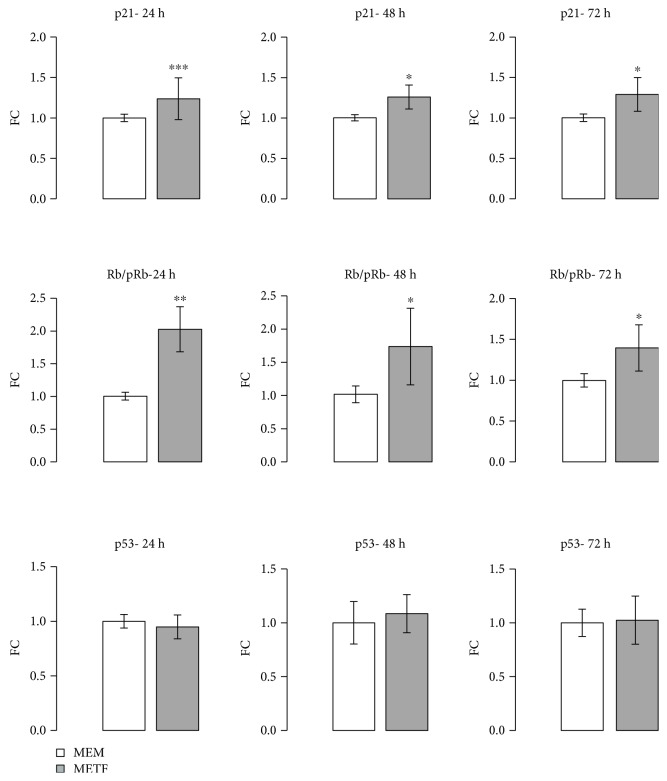
METF action on cell cycle protein: METF significantly increased p21 and Rb protein content at 24, 48, and 72 h of treatment in respect to MEM control, without modification of the p53 protein level. Data are expressed as fold change (FC) mean ± SD. Representative Western blots were added as supplementary data. Significance: *t*-test: ^∗^ *p* ≤ 0.05 vs MEM, ^∗∗^ *p* ≤ 0.01 vs MEM, and ^∗∗∗^ *p* ≤ 0.001 vs MEM.

**Figure 4:**
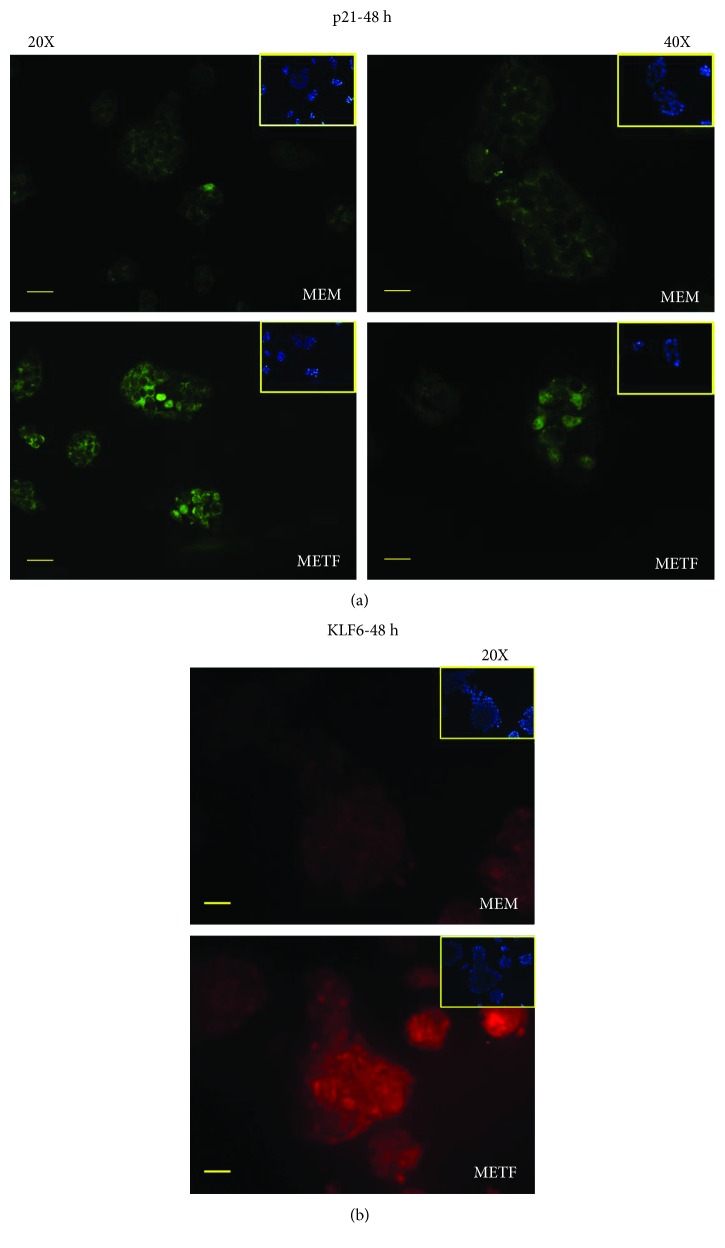
METF action on antiproliferative marker expression: (a) immunofluorescence data indicated that METF enhanced p21 nuclear translocation, confirming that METF was able to influence the key regulators of cell cycle; (b) immunofluorescence assay showed that METF improved KLF6 protein content after 48 h of treatment. Scale bars: 200 *μ*m (20X) and 100 *μ*m (40X).

A similar trend was also observed when HepG2 cells have reached 80% confluence: in this phase, the Rb/pRb ratio and p21 protein content were improved at all time points after the addition of METF, regardless of p53 levels (Figure 3). Additionally, p21 nuclei localization was confirmed by immunofluorescence assay after 48 hours of treatment (Figure 4(a)).

Kruppel-like factor 6 (KLF6) is a transcriptional factor which plays roles in human tumorigenesis [35]: in particular, KLF6 enhances p21 expression independently from p53 [36, 37]. KLF6 was raised after 48 hours of treatment with METF (Figure 4(b)).

### 3.4. Metformin and AMPK

**Figure 5:**
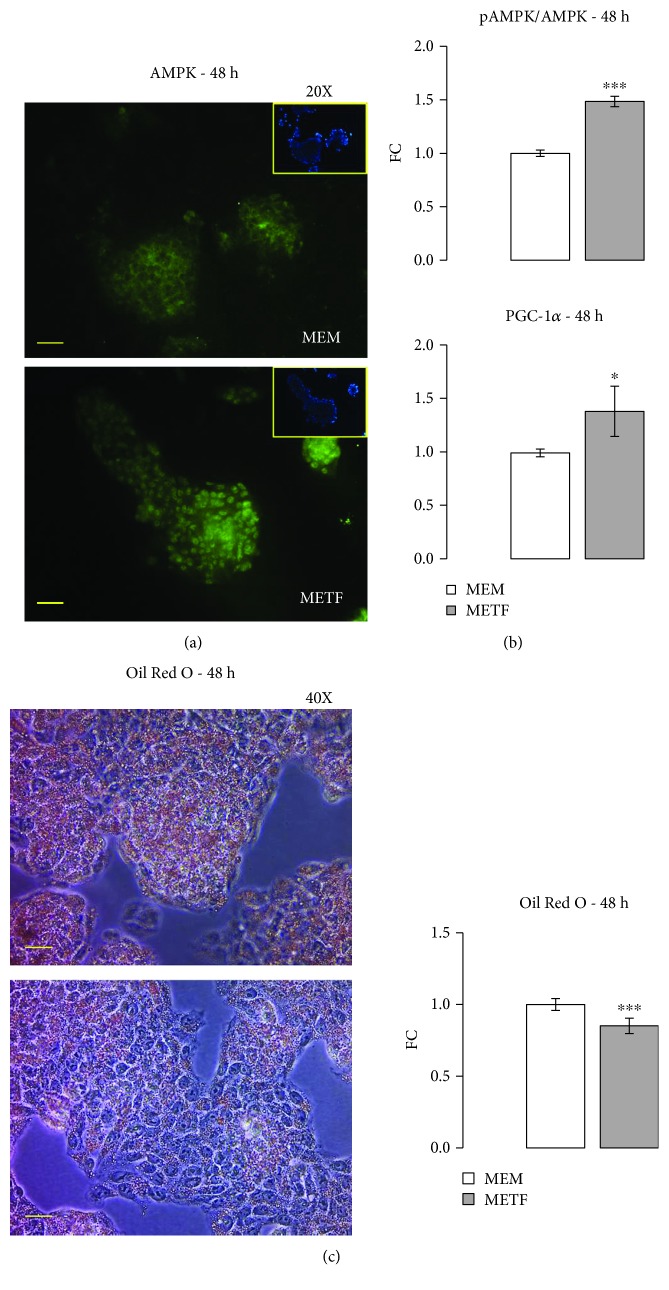
METF action on lipid metabolism: (a) immunofluorescence assay confirmed the increase of AMPK protein expression with respect to control at 48 h of treatment; (b) Western blot results pointed out that METF significantly increased the pAMPK/AMPK ratio and PGC-1*α* protein level; (c) Oil Red O coloration and relevant quantification revealed that METF decreased lipid accumulation in HepG2. Data are expressed as fold change (FC) mean ± SD. Representative Western blots were added as supplementary data. Significance: *t*-test: ^∗^ *p* ≤ 0.05 vs MEM and ^∗∗∗^ *p* ≤ 0.001 vs MEM. Scale bars: 200 *μ*m (20X) and 100 *μ*m (40X).

To detect whether metformin affects AMPK protein synthesis in HepG2 cells, AMPK expression was measured by immunofluorescence assay. The data confirmed that metformin increases the kinase's protein expression (Figure 5(a)) and its activation (Figure 5(b)), as shown by Western blot assay. AMPK might play an important role in metformin-induced growth inhibition.

Since AMPK inhibits limiting steps in lipogenesis, leading to decreased lipid deposition [19, 38], metformin could improve hepatic steatosis by increasing its phosphorylation. The effects of metformin on lipolysis were confirmed by Western blotting for peroxisome proliferator-activated receptor-*γ* coactivator-1*α* (PGC-1*α*), a transcriptional coactivator that has emerged as a master regulator of hepatic energy metabolism [39]: 400 *μ*M METF increases the protein content of PGC-1*α* (Figure 5(b)). Through various interactions, PGC-1*α* plays an important role in fatty acid oxidation [40] and gluconeogenesis in the liver [41].

Above all, Oil Red O staining showed that intracellular lipid deposition significantly decreased with 400 *μ*M metformin (Figure 5(c)).

### 3.5. Metformin Inhibits the Invasive Potential of HCC Cells In Vitro

The anticancer effects of METF are associated to cell cycle arrest, induction of apoptosis, and inhibition of metastasis invasion. For this reason, we investigated the action of 400 *μ*M METF on the main markers of liver tumorigenesis.

**Figure 6:**
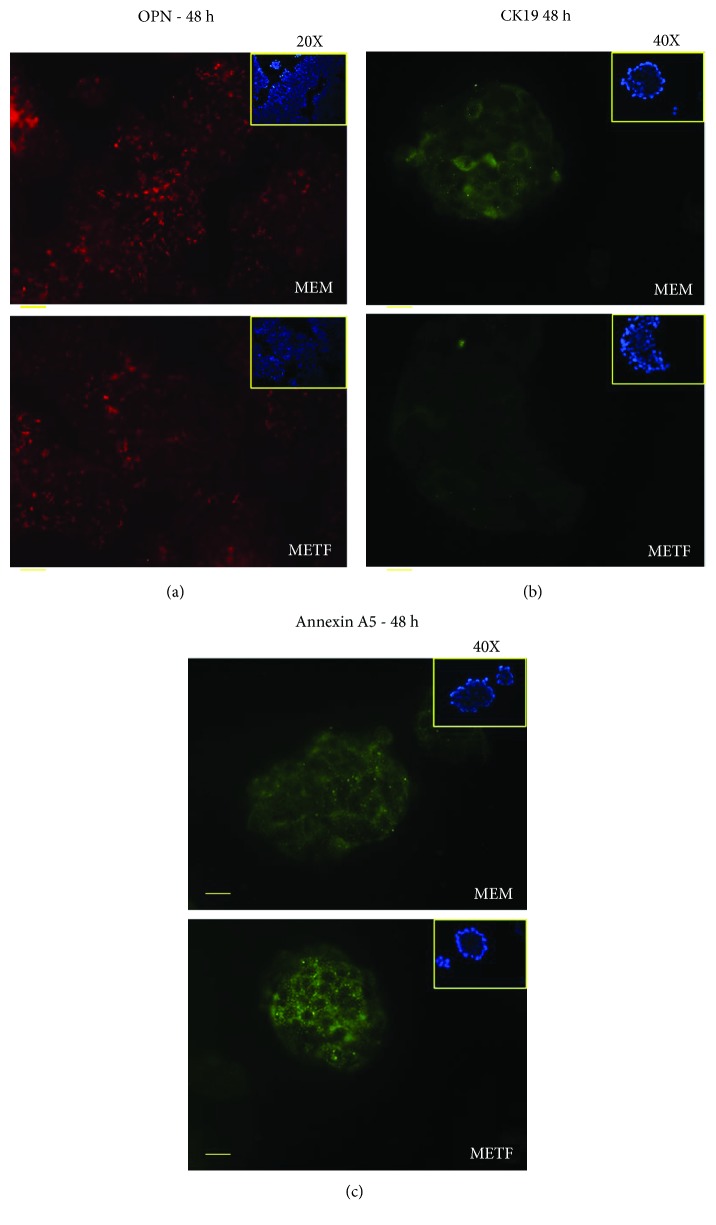
METF action on tumorigenesis marker expression: (a) after 48 h of differentiation, METF treatment could decrease OPN protein expression compared with MEM control; (b) immunofluorescence assay of CK19 after 48 h of treatment showed how METF could decrease CK19 protein expression in respect to MEM control; (c) immunofluorescence assay for annexin A5 after 48 h of treatment revealed that METF was able to increase protein expression in respect to MEM control. Scale bars: 200 *μ*m (20X) and 100 *μ*m (40X).

Cytokine osteopontin (OPN), one of the metastatic genes, was upregulated in HCC, and the increase of its expression was correlated with the metastatic ability of HCC and invasiveness of liver tumor-derived cell lines *in vitro* [11, 42]. Recently, Zhu et al. have demonstrated that OPN enhances CCR1 expression, an important chemokine receptor, through the PI3K/AKT/HIF-1a signaling pathway. The CCR1 receptor plays a key role in promoting HCC metastasis. As shown in Figure 6(a), the OPN protein content in HepG2 cells, which express constitutively high levels of the protein [11], was decreased after 48 hours of drug treatment.

Cytokeratin 19 (CK19) positivity in HCC cells was well correlated with the clinical and pathological features of tumor aggressiveness and poor prognosis [43, 44]. Moreover, CK19 positivity in HCC was associated with increased expression of epithelial-mesenchymal transition- (EMT-) related genes and invasion-related proteins [8]. METF inhibits the expression of this marker after 48 hours of treatment (Figure 6(b)).

Therefore, the data collected indicate that METF may influence adversely markers of tumorigenesis, such as OPN and CK19, and constrain the cell migration and invasion in liver cancer cells.

Considering the new role of annexin A5 in cancer therapy [12], we analyzed the action of METF on annexin A5 by immunofluorescence assay. As shown in Figure 6(c), after 48 h of treatment, METF increased annexin A5 protein content.

### 3.6. Metformin Role on the IGF Axis

The insulin-like growth factor (IGF) signalling pathway is another important pathway in the process of hepatocarcinogenesis: three IGF axis members are known to be involved in the development of HCC [45].

In 16%–40% of human HCC, IGF-II overexpression has been evidenced [46]. Experimental induction of IGF-II expression was positively correlated with enhanced cell growth and tumor neovascularization; moreover, its inhibition promoted apoptosis [47, 48]. Healthy hepatocytes do not express IGF-IR, whereas HCC cells exhibit overexpression and overactivation of the IGF-I receptor [49]. Rodriguez-Tarduchy et al. reported that the primary tumorigenic effects of IGFs are regulated by IGF-IR [50].

**Figure 7:**
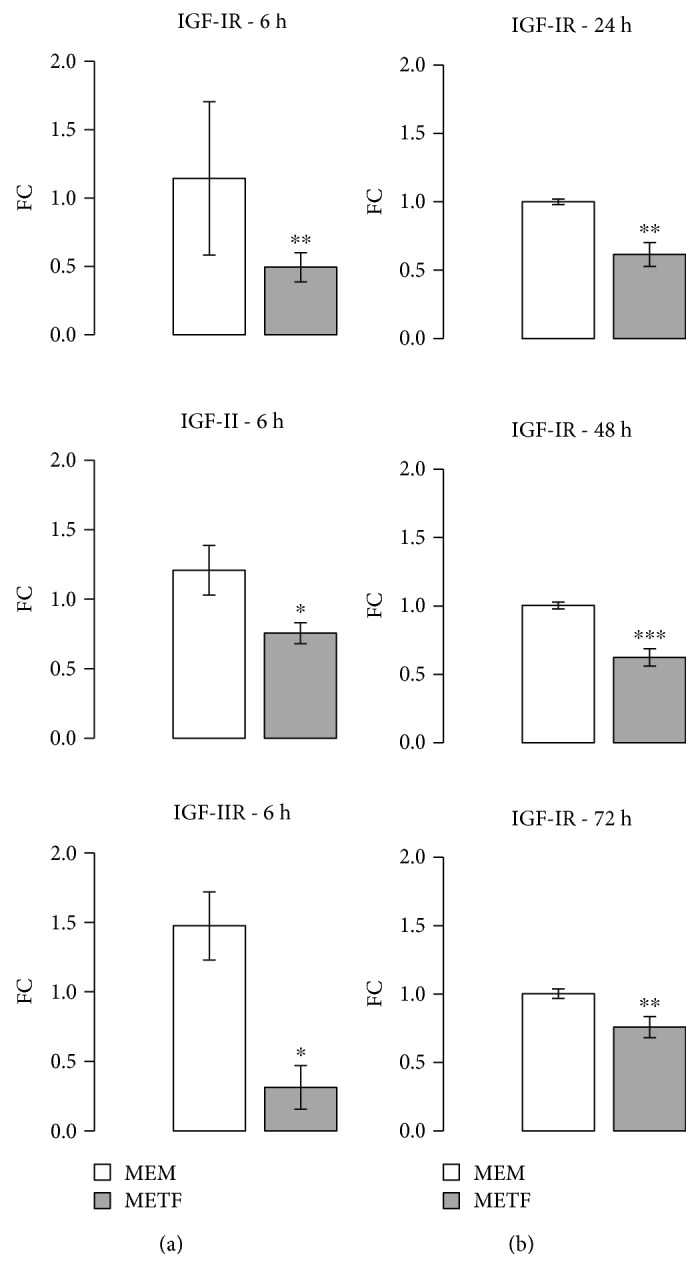
METF action on IGFs axis: (a) real-time PCR results showed that METF significantly decreased IGF-II and IGF-II R expression; (b) Western blot data indicated that METF significantly reduced IGF-I R protein content. Data are expressed as fold change (FC) mean ± SD. Representative Western blots were added as supplementary data. Significance: *t*-test: ^∗^ *p* ≤ 0.05 vs MEM, ^∗∗^ *p* ≤ 0.01 vs MEM, and ^∗∗∗^ *p* ≤ 0.001 vs MEM.

We decided to analyze these three IGF axis members in HepG2 cells treated with METF. Real-time PCR data showed that after 6 hours of treatment with 400 *μ*M METF, IGF-II/IGF-IIR gene expression was significantly reduced (Figure 7(a)). Furthermore, HepG2 cells exposed to METF produced significantly less IGF-IR at all time points (Figure 7(b)).

## 4. Discussion

HCC is a type of malignant cancer associated with a high incidence and rate of mortality. Nowadays, the therapeutic approaches available for the treatment of HCC are insufficient.

Recent data suggest that metformin could protect patients with type II diabetes from cancer [22] and, in particular, it could inhibit liver cancer cell proliferation *in vitro* and *in vivo* [28, 31]. The HCC risk in these patients was found to be as high as 7.1 higher than that in nondiabetic patients, depending on the duration of diabetes and the protocol used in the treatment [27, 51].

Over two hundred clinical trials about metformin as anticancer drug, completed or in progress, have not yet registered positive clinical results. Probably, the major obstacle of these unsatisfactory results is the concentration of metformin on target organs [52].

The maximum concentration of drug in the circulating system is less than 60 *μ*M via oral administration, much less than 2 mM, the minimum effective antitumor concentration suggested in *in vitro* works [53]. In addition, increase of the oral dose of metformin is not the right solution because you have more side effects, as gastrointestinal discomfort and lactic acidosis, due to the pharmacokinetics of drug [52]. For these reasons, we performed our experiments using a 400 *μ*M metformin dose, which corresponds to the *in vitro* human therapeutic dose.

Previous studies have revealed that metformin is able to inhibit HCC growth through regulation of the AMPK-dependent pathway [31]. However, Xiong et al. showed that the induction of HCC cell cycle arrest and apoptosis by metformin was through an AMPK-independent pathway [20, 21, 54]. Indeed, AMPK can also influence the metabolism of fatty acids [19], whose alterations are mainly responsible for the inflammatory microenvironment favouring the development of the same HCC [55]. In this study, we observed that 400 *μ*M metformin significantly increased AMPK phosphorylation on Thr 172 and is able to decrease the accumulation of intracellular lipids (Figure 5) Therefore, metformin, and other molecules that target AMPK, may play a dual action, both as antiproliferative agent and as agents able to counteract the inflammatory microenvironment.

In liver tumor cells, the invasiveness, the intrahepatic dissemination, and metastasis generate a high level of aggressiveness. Clinical and experimental evidence, regarding the link between OPN and HCC metastasis, makes OPN an attractive potential therapeutic target against HCC metastasis [56]. OPN is markedly elevated in the plasma of HCC patients, [57] and, when neutralized with antibody, it inhibits the *in vitro* invasion and *in vivo* lung metastasis of highly metastatic HCC cells [58]. Using immunofluorescence assay, we found that 400 *μ*M of METF decreased expression levels of OPN in HepG2 (Figure 6(a)).

Our results show that 400 *μ*M metformin is the sufficient dose to decrease HepG2 cell proliferation through the arrest in the G0/G1 phase of the cell cycle, without cell death (Figures 1 [Fig fig2]–3). Many studies performed in liver cancer cells have shown that metformin selectively induces or enhances apoptosis [30] but only at high concentrations, incompatible with the human health. Conversely, several studies demonstrated that metformin protects against apoptosis in normal cells [29].

The phosphorylation of Rb plays a crucial role in the progression of the G1 phase and the transition of the G1 to the S phase [33, 59, 60]. Our results (Figures 2 and 3) suggest that treatment with 400 *μ*M metformin increased the protein content of p21, resulting in hypophosphorylation of Rb, which arrests the cell at the G1 phase [57, 59, 60].

Indeed, our data show that the increase in p21 expression levels after metformin treatment (Figure 4(a)) is not due to the action of p53 but to KLF6. KLF6 is frequently inactivated in HCC [35] and, when overexpressed in carcinoma-derived cells, interacts with cyclin D to disrupt cyclin/CDK complexes, to redistribute p21 to CDK2, which promotes G1 cell cycle arrest [61]. Furthermore, it has been showed that the silencing of KLF6 induces death cell by apoptosis through p53 upregulation and the inhibition of Bcl-xL expression: this shows that KLF6, increasing the level of p21 protein by the p53 protein, is essential for liver cancer cells to evade apoptosis. Our results on the KLF6/p21/Rb axis contribute to explain the growth inhibitory effect of 400 *μ*M metformin on the human hepatic cancer HepG2.

The effect of 400 *μ*M metformin on CK19 protein seems to be very interesting above all those related to Sorafenib. This drug, an orally available kinase inhibitor, is the only standard clinical treatment against advanced HCC. However, many patients show drug resistance. Increasing evidence suggests that this phenomenon in HCC is correlated with the activation of epithelial-mesenchymal transition (EMT) and enrichment of cancer stem cell (CSC) traits [62]. Increased expression of CK19 is related to these events. Metformin, acting on CK19 synthesis, could increase the sensitivity of HCC cells to Sorafenib and inhibit HCC recurrence and metastasis [63]. In support of this our speculation, Kang et al. showed that the combined administration of metformin and Sorafenib significantly inhibits the recurrence and metastasis of primary liver cancer in HCC patients, after surgical resection [64]. Moreover, we observed that METF stimuli increased annexin A5 protein expression (Figure 6(c)): recent data indicate how the upregulation of this protein ameliorates antitumoral response, acting on cell cycle regulators [12, 13]. We speculated that METF, acting on annexin A5, could represent a novel coadjuvant in cancer therapy.

Molecular target therapy is now developing as a novel anticancer modality and seems to be a promising way for prolonging advanced HCC patient survival. Insulin-like growth factor (IGF) signaling is specifically required for hepatocyte malignant transformation and HCC progression [65, 66], especially the IGF-I receptor (IGF-IR) [13] and IGF-II expression in hepatocarcinogenesis [46, 65]. IGF-IR stimulates growth of HCC cells through the activation of the IGF-II/IGF-IR pathway: this receptor is not expressed in healthy mature hepatocytes, whereas HCC cells exhibit abnormal activation. These observations are in agreement with previous data exhibiting that IGF-I or IGF-II is higher in tumors over other liver tissues [67]. IGF-II is overexpressed at the early stage of HCC [46, 68] and mediated cell proliferation mainly via a transmembrane tyrosine kinase by a paracrine mechanism then leads to activation of the phosphatidylinositol 3-kinase- and Ras/mitogen-activated protein kinase pathways [69]. Yao et al. confirmed that the IGF-IR gene silencing through the IGF-II/IGF-IR pathway is one of the molecular mechanisms that inhibited HCC cell proliferation because its activation is likely a progression switch selected by function that promotes HCC dissemination [70, 71]. Interfering with signaling via IGF-IR has an antitumor effect by inhibiting cell growth. Hypoxia inducible factors upregulate IGF-II that, in turn, promotes VEGFA expression leading to angiogenesis [72]. Moreover, it has been reported that blockage of IGF-II expression causes downregulation of VEGFA and inhibits growth in HCC cells [70]. Altogether, these results suggest that IGF-II might play a role in regulating tumor angiogenesis in HCC. We showed that 400 *μ*M of METF is able to significantly decrease not only IGF-IR level expression but also the expression of the IGF2-II/IGF-IIR genes, already after 6 hours of treatment (Figure 7). The present data confirmed that acting on IGF-IR should have effect on the biological functions of HCC cells *in vitro* through the IGF-II/IGF-IR pathway, suggesting that the IGF-IR pathway should be a novel therapeutic target for HCC.

## 5. Conclusion

The data of this study take particular importance since it is the first work in which the dose of 400 *μ*M metformin showed antiproliferative effects on HCC cells: in fact, previous works used a high dose of metformin with dangerous effects. Interestingly, our *in vitro* data show that 400 *μ*M metformin decreases cell proliferation, indicating how this dose could be sufficient to exhibit anticancer effects. Of course, this dose of the drug may not be considered curative but a great adjuvant in current therapies for the treatment of HCC. METF, in this dose, could represent an interesting and promising adjuvant in traditional and new therapies for the treatment of HCC, i.e., radiotherapy.

Further studies should be performed in order to investigate METF clinical application and its combination with other chemotherapy.

## Data Availability

The data used to support the findings of this study are available from the corresponding author upon request.

## Supplementary Materials

Supplementary 1 Figure 1S: immunofluorescence images obtained by BrdU assay (Figure 1-graph C). Western blot images relative to p53, p21, and pRb/Rb during the proliferation phase of HepG2 treated with metformin (Figure 1-graphs D-E).

Supplementary 2 Figure 2S: Western blot images relative to p53, p21, and pRb/Rb (Figure 3). Western blot images relative to pAMPK/AMPK and PGC-1*α* (Figure 5-graph B). Western blot images relative to IGFI-R (Figure 7-graph B).

## References

[1] Dhanasekaran R., Limaye A., Cabrera R. (2012). Hepatocellular carcinoma: current trends in worldwide epidemiology, risk factors, diagnosis, and therapeutics. *Hepatic Medicine: Evidence and Research*.

[2] Wang C., Wang X., Gong G. (2012). Increased risk of hepatocellular carcinoma in patients with diabetes mellitus: a systematic review and meta-analysis of cohort studies. *International Journal of Cancer*.

[3] Stravitz R. T., Heuman D. M., Chand N. (2008). Surveillance for hepatocellular carcinoma in patients with cirrhosis improves outcome. *The American Journal of Medicine*.

[4] Katyal S., Oliver J. H., Peterson M. S., Ferris J. V., Carr B. S., Baron R. L. (2000). Extrahepatic metastases of hepatocellular carcinoma. *Radiology*.

[5] Valastyan S., Weinberg R. A. (2011). Tumor metastasis: molecular insights and evolving paradigms. *Cell*.

[6] Quail D. F., Joyce J. A. (2013). Microenvironmental regulation of tumor progression and metastasis. *Nature Medicine*.

[7] Govaere O., Komuta M., Berkers J. (2014). Keratin 19: a key role player in the invasion of human hepatocellular carcinomas. *Gut*.

[8] Wang H., Chen L. (2013). Tumor microenviroment and hepatocellular carcinoma metastasis. *Journal of Gastroenterology and Hepatology*.

[9] Li H., Zhang L. (2017). Liver regeneration microenvironment of hepatocellular carcinoma for prevention and therapy. *Oncotarget*.

[10] Wai P. Y., Kuo P. C. (2008). Osteopontin: regulation in tumor metastasis. *Cancer Metastasis Reviews*.

[11] Dong Q., Zhu X., Dai C. (2016). Osteopontin promotes epithelial_mesenchymal transition of hepatocellular carcinoma through regulating vimentin. *Oncotarget*.

[12] Jeong J. J., Park N., Kwon Y. J., Ye D. J., Moon A., Chun Y. J. (2014). Role of annexin A5 in cisplatin-induced toxicity in renal cells: molecular mechanism of apoptosis. *Journal of Biological Chemistry*.

[13] Li X., Ma W., Wang X., Ci Y., Zhao Y. (2018). Annexin A5 overexpression might suppress proliferation and metastasis of human uterine cervical carcinoma cells. *Cancer Biomarkers*.

[14] Baserga R. (1999). The IGF-I receptor in cancer research. *Experimental Cell Research*.

[15] Senesi P., Montesano A., Luzi L., Codella R., Benedini S., Terruzzi I. (2016). Metformin treatment prevents sedentariness related damages in mice. *Journal Diabetes Research*.

[16] Shaw R., Lamia K., Vasquez D. (2005). The kinase LKB1 mediates glucose homeostasis in liver and therapeutic effects of metformin. *Science*.

[17] Hawley S. A., Gadalla A. E., Olsen G. S., Hardie D. G. (2002). The antidiabetic drug metformin activates the AMP-activated protein kinase cascade via an adenine nucleotide-independent mechanism. *Diabetes*.

[18] Lv Q., Zhen Q., Liu L. (2015). AMP-kinase pathway is involved in tumor necrosis factor alpha-induced lipid accumulation in human hepatoma cells. *Life Sciences*.

[19] Hardie D. G. (2014). AMPK-sensing energy while talking to other signaling pathways. *Cell Metabolism*.

[20] Kalender A., Selvaraj A., Kim S. Y. (2010). Metformin, independent of AMPK, inhibits mTORC1 in a rag GTPase-dependent manner. *Cell Metabolism*.

[21] Madiraju A. K., Erion D. M., Rahimi Y. (2014). Metformin suppresses gluconeogenesis by inhibiting mitochondrial glycerophosphate dehydrogenase. *Nature*.

[22] Zhang H., Gao C., Fang L., Zhao H. C., Yao S. K. (2013). Metformin and reduced risk of hepatocellular carcinoma in diabetic patients: a meta-analysis. *Scandinavian Journal of Gastroenterology*.

[23] Dowling R. J. O., Niraula S., Stambolic V., Goodwin P. J. (2012). Metformin in cancer: translational challenges. *Journal of Molecular Endocrinology*.

[24] Gallagher E. J., LeRoith D. (2011). Diabetes, cancer, and metformin: connections of metabolism and cell proliferation. *Annals of the New York Academy of Sciences*.

[25] Bhalla K., Hwang B. J., Dewi R. E. (2012). Metformin prevents liver tumorigenesis by inhibiting pathways driving hepatic lipogenesis. *Cancer Prevention Research*.

[26] Singh S., Singh P. P., Singh A. G., Murad M. H., Sanchez W. (2013). Anti-diabetic medications and the risk of hepatocellular cancer: a systematic review and meta-analysis. *The American Journal of Gastroenterology*.

[27] Chang C. H., Lin J. W., Wu L. C., Lai M. S., Chuang L. M. (2012). Oral insulin secretagogues, insulin, and cancer risk in type 2 diabetes mellitus. *The Journal of Clinical Endocrinology & Metabolism*.

[28] Qu Z., Zhang Y., Liao M., Chen Y., Zhao J., Pan Y. (2012). In vitro and in vivo antitumoral action of metformin on hepatocellular carcinoma. *Hepatology Research*.

[29] Miyoshi H., Kato K., Iwama H. (2014). Effect of the anti-diabetic drug metformin in hepatocellular carcinoma in vitro and in vivo. *International Journal of Oncology*.

[30] Cai X., Hu X., Cai B. (2013). Metformin suppresses hepatocellular carcinoma cell growth through induction of cell cycle G1/G0 phase arrest and p21CIP and p27KIP expression and downregulation of cyclin D1 in vitro and in vivo. *Oncology Reports*.

[31] Cheng J., Huang T., Li Y. (2014). AMP-activated protein kinase suppresses the *in vitro* and *in vivo* proliferation of hepatocellular carcinoma. *PLoS One*.

[32] Xiong Y., Lu Q. J., Zhao J., Wu G. Y. (2012). Metformin inhibits growth of hepatocellular carcinoma cells by inducing apoptosis via mitochondrion-mediated pathway. *Asian Pacific Journal of Cancer Prevention*.

[33] Vermeulen K., Berneman Z. N., Van Bockstaele D. R. (2003). Cell cycle and apoptosis. *Cell Proliferation*.

[34] Ileana T., Anna M., Pamela S., Fernanda V., Stefano B., Livio L. (2017). Ranolazine promotes muscle differentiation and reduces oxidative stress in C2C12 skeletal muscle cells. *Endocrine*.

[35] Kremer-Tal S., Reeves H. L., Narla G. (2004). Frequent inactivation of the tumor suppressor Kruppel-like factor 6 (KLF6) in hepatocellular carcinoma. *Hepatology*.

[36] Narla G., Kremer-Tal S., Matsumoto N. (2007). In vivo regulation of p 21 by the Kruppel-like factor 6 tumor-suppressor gene in mouse liver and human hepatocellular carcinoma. *Oncogene*.

[37] Abbas T., Dutta A. (2009). P 21 in cancer: intricate networks and multiple activities. *Nature Reviews Cancer*.

[38] Zhu X., Yan H., Xia M. (2018). Metformin attenuates triglyceride accumulation in HepG2 cells through decreasing stearyl-coenzyme A desaturase 1 expression. *Lipids in Health and Disease*.

[39] Finck B., Kelly D. P. (2006). PGC-1 coactivators: inducible regulators of energy metabolism in health and disease. *Journal of Clinical Investigation*.

[40] Zhang Y., Castellani L. W., Sinal C. J., Gonzalez F. J., Edwards P. A. (2004). Peroxisome proliferator-activated receptor-*γ* coactivator 1*α* (PGC-1*α*) regulates triglyceride metabolism by activation of the nuclear receptor FXR. *Genes & Development*.

[41] Herzig S., Long F., Jhala U. S. (2001). CREB regulates hepatic gluconeogenesis through the coactivator PGC-1. *Nature*.

[42] Zhu Y., Gao X.-M., Yang J. (2018). C-C chemokine receptor type 1 mediates osteopontin-promoted metastasis in hepatocellular carcinoma. *Cancer Science*.

[43] Kim H., Choi G. H., Na D. C. (2011). Human hepatocellular carcinomas with “stemness”-related marker expression: keratin 19 expression and a poor prognosis. *Hepatology*.

[44] Romano M., Francesco F. D., Pirozzi G. (2015). Expression of cancer stem cell biomarkers as a tool for a correct therapeutic approach to hepatocellular carcinoma. *Oncoscience*.

[45] El Tayebi H. M., Abdelaziz A. I. (2016). Epigenetic regulation of insulin-like growth factor axis in hepatocellular carcinoma. *World Journal of Gastroenterology*.

[46] Cariani E., Lasserre C., Seurin D. (1988). Differential expression of insulin-like growth factor II mRNA in human primary liver cancers, benign liver tumors, and liver cirrhosis. *Cancer Research*.

[47] Lund P., Schubert D., Niketeghad F., Schirmacher P. (2004). Autocrine inhibition of chemotherapy response in human liver tumor cells by insulin-like growth factor-II. *Cancer Letters*.

[48] Yao X., Hu J. F., Daniels M. (2003). A methylated oligonucleotide inhibits IGF2 expression and enhances survival in a model of hepatocellular carcinoma. *The Journal of Clinical Investigation*.

[49] Aleem E., Nehrbass D., Klimek F., Mayer D., Bannasch P. (2011). Upregulation of the insulin receptor and type I insulin-like growth factor receptor are early events in hepatocarcinogenesis. *Toxicologic Pathology*.

[50] Rodriguez-Tarduchy G., Collins M. K., García I., López-Rivas A. (1992). Insulin-like growth factor-I inhibits apoptosis in IL-3-dependent hemopoietic cells. *The Journal of Immunology*.

[51] Lai S. W., Chen P. C., Liao K. F., Muo C. H., Lin C. C., Sung F. C. (2012). Risk of hepatocellular carcinoma in diabetic patients and risk reduction associated with anti-diabetic therapy: a population-based cohort study. *The American Journal of Gastroenterology*.

[52] Menendez J. A., Quirantes-Piné R., Rodríguez-Gallego E. (2014). Oncobiguanides: Paracelsus' law and nonconventional routes for administering diabetobiguanides for cancer treatment. *Oncotarget*.

[53] Xiao Z., Sperl B., Ullrich A., Knyazev P. (2014). Metformin and salinomycin as the best combination for the eradication of NSCLC monolayer cells and their alveospheres (cancer stem cells) irrespective of EGFR, KRAS, EML4/ALK and LKB1 status. *Oncotarget*.

[54] Bhat M., Yanagiya A., Graber T. (2016). Metformin requires 4E-BPs to induce apoptosis and repress translation of Mcl-1 in hepatocellular carcinoma cells. *Oncotarget*.

[55] Cauchy F., Mebarki M., Leporq B. (2016). Strong antineoplastic effects of metformin in preclinical models of liver carcinogenesis. *Clinical Science*.

[56] Shang S., Plymoth A., Ge S. (2012). Identification of osteopontin as a novel marker for early hepatocellular carcinoma. *Hepatology*.

[57] Kim J., Ki S. S., Lee S. D. (2006). Elevated plasma osteopontin levels in patients with hepatocellular carcinoma. *The American Journal of Gastroenterology*.

[58] Ye Q. H., Qin L. X., Forgues M. (2003). Predicting hepatitis B virus-positive metastatic hepatocellular carcinomas using gene expression profiling and supervised machine learning. *Nature Medicine*.

[59] Liang X., Wang P., Gao Q., Tao X. (2014). Exogenous activation of LKB1/AMPK signaling induces G1 arrest in cells with endogenous LKB1 expression. *Molecular Medicine Reports*.

[60] Massagué J. (2004). G1 cell-cycle control and cancer. *Nature*.

[61] Benzeno S., Narla G., Allina J. (2004). Cyclin-dependent kinase inhibition by the KLF6 tumor suppressor protein through interaction with cyclin D1. *Cancer Research*.

[62] Kawai T., Yasuchika K., Ishii T. (2015). Keratin 19, a cancer stem cell marker in human hepatocellular carcinoma. *Clinical Cancer Research*.

[63] Chen J., Jin R., Zhao J. (2015). Potential molecular, cellular and microenvironmental mechanism of sorafenib resistance in hepatocellular carcinoma. *Cancer Letters*.

[64] Kang W. H., Tak E., Hwang S. (2018). Metformin-associated chemopreventive effects on recurrence after hepatic resection of hepatocellular carcinoma: from in vitro to a clinical study. *Anticancer Research*.

[65] Nussbaum T., Samarin J., Ehemann V. (2008). Autocrine insulin-like growth factor-II stimulation of tumor cell migration is a progression step in human hepatocarcinogenesis. *Hepatology*.

[66] Chen Y. W., Boyartchuk V., Lewis B. C. (2009). Differential roles of insulin-like growth factor receptor- and insulin receptor-mediated signaling in the phenotypes of hepatocellular carcinoma cells. *Neoplasia*.

[67] El Tayebi H. M., Salah W., El Sayed I. H. (2011). Expression of insulin-like growth factor-II, matrix metalloproteinases, and their tissue inhibitors as predictive markers in the peripheral blood of HCC patients. *Biomarkers*.

[68] Martinez-Quetglas I., Pinyol R., Dauch D. (2016). IGF2 is upregulated by epigenetic mechanisms in hepatocellular carcinomas and is an actionable oncogene product in experimental models. *Gastroenterology*.

[69] Alexia C., Bras M., Fallot G. (2006). Pleiotropic effects of PI-3′ kinase/Akt signaling in human hepatoma cell proliferation and drug-induced apoptosis. *Annals of the New York Academy of Sciences*.

[70] Yao N., Yao D., Wang L. (2012). Inhibition of autocrine IGF-II on effect of human HepG2 cell proliferation and angiogenesis factor expression. *Tumour Biology*.

[71] Yao M., Wang L., Yang J., Yan X., Cai Y., Yao D. (2016). IGF-I receptor as an emerging potential molecular-targeted for hepatocellular carcinoma *in vitro* and *in vivo*. *Tumour Biology*.

[72] Kim K. W., Bae S. K., Lee O. H., Bae M. H., Lee M. J., Park B. C. (1998). Insulin-like growth factor II induced by hypoxia may contribute to angiogenesis of human hepatocellular carcinoma. *Cancer Research*.

