# Supplementary figures and images for: Metformin Counteracts HCC Progression and Metastasis Enhancing KLF6/p21 Expression and Downregulating the IGF Axis
Source: Int J Endocrinol. 2019 Jan 10;2019:7570146. doi: 10.1155/2019/7570146 (PMC6350585; doi:10.1155/2019/7570146)

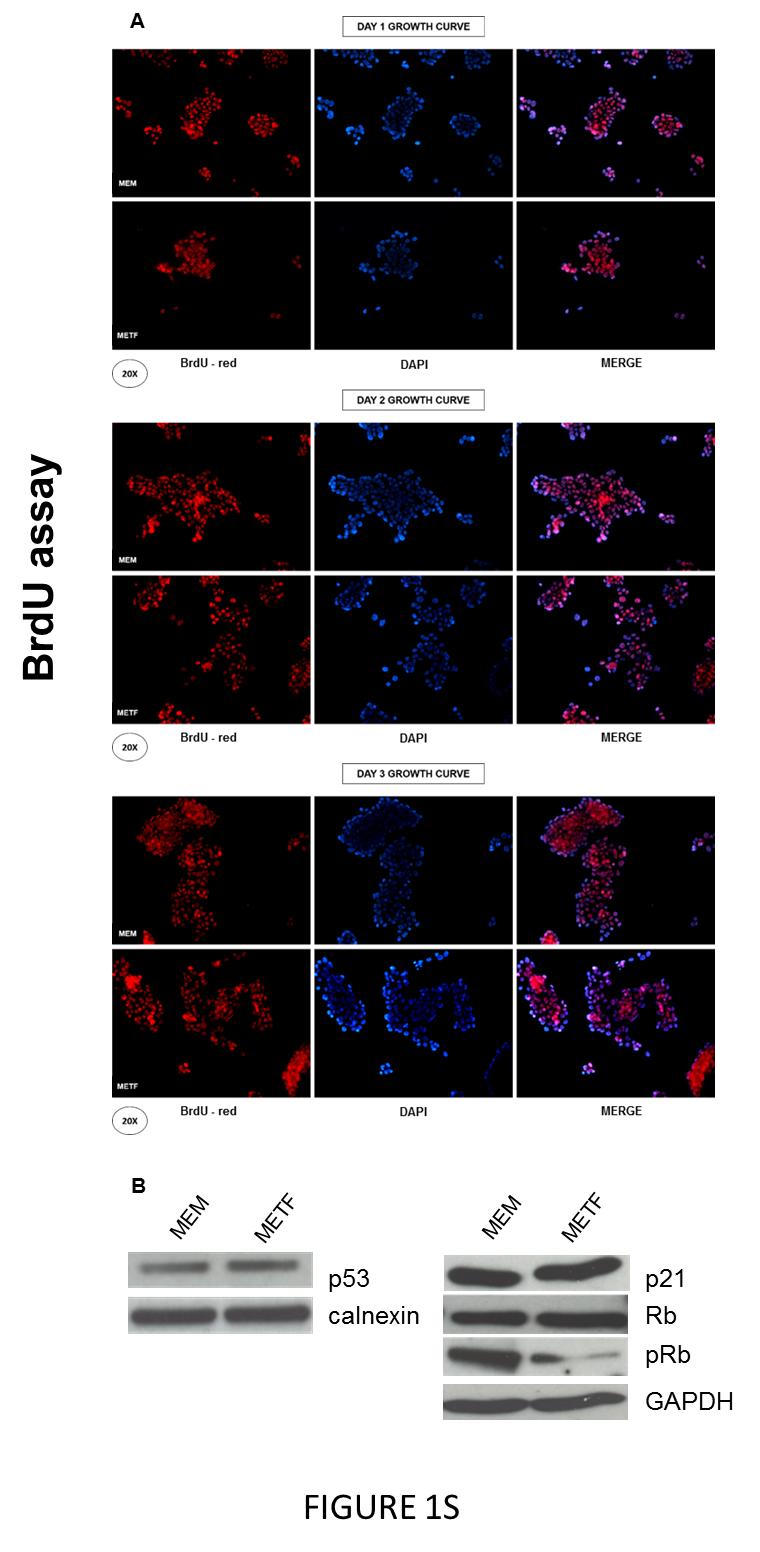

Supplement: Supplementary 1 — Figure 1S: immunofluorescence images obtained by BrdU assay (Figure 1-graph C). Western blot images relative to p53, p21, and pRb/Rb during the proliferation phase of HepG2 treated with metformin (Figure 1-graphs D-E). [file 7570146.f1.docx]

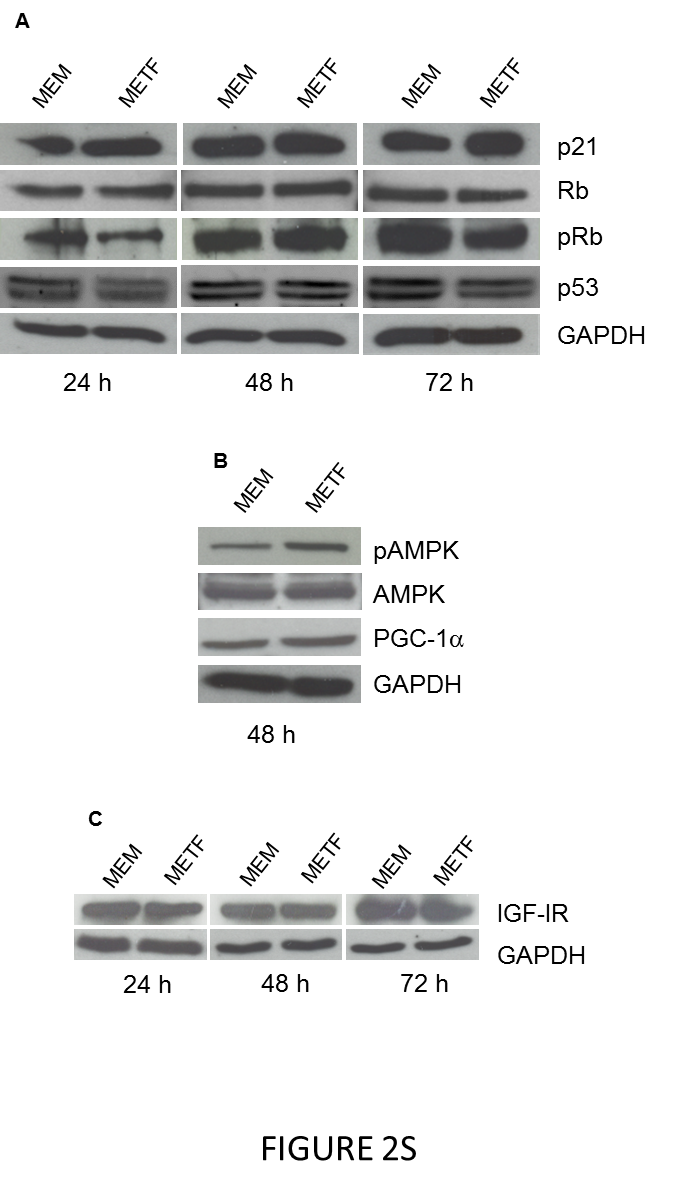

Supplement: Supplementary 2 — Figure 2S: Western blot images relative to p53, p21, and pRb/Rb (Figure 3). Western blot images relative to pAMPK/AMPK and PGC-1α (Figure 5-graph B). Western blot images relative to IGFI-R (Figure 7-graph B). [file 7570146.f2.docx]
